# Oral administration of the cannabigerol derivative VCE-003.2 promotes subventricular zone neurogenesis and protects against mutant huntingtin-induced neurodegeneration

**DOI:** 10.1186/s40035-019-0148-x

**Published:** 2019-03-08

**Authors:** José Aguareles, Juan Paraíso-Luna, Belén Palomares, Raquel Bajo-Grañeras, Carmen Navarrete, Andrea Ruiz-Calvo, Daniel García-Rincón, Elena García-Taboada, Manuel Guzmán, Eduardo Muñoz, Ismael Galve-Roperh

**Affiliations:** 1 Instituto Ramón y Cajal de Investigación Sanitaria (IRYCIS), Ctra. Colmenar Viejo, km, 9100 Madrid, Spain; 2 Departamento de Bioquímica y Biología Molecular and Instituto Universitario de Investigación Neuroquímica, Universidad Complutense, Madrid, Spain; 3 Centro de Investigación Biomédica en Red sobre Enfermedades Neurodegenerativas (CIBERNED), Madrid, Spain; 4 Instituto Maimónides de Investigación Biomédica de Córdoba (IMIBIC), Córdoba, Spain; 5 Departamento de Biología Celular, Fisiología e Inmunología, Universidad de Córdoba, Córdoba, Spain; 6 Hospital Universitario Reina Sofía, Córdoba, Spain; 7 Emerald Health Pharmaceuticals, San Diego, USA

**Keywords:** Cannabinoid, Huntingtin, Neurodegeneration, Neurogenesis, PPAR

## Abstract

**Background:**

The administration of certain cannabinoids provides neuroprotection in models of neurodegenerative diseases by acting through various cellular and molecular mechanisms. Many cannabinoid actions in the nervous system are mediated by CB_1_ receptors, which can elicit psychotropic effects, but other targets devoid of psychotropic activity, including CB_2_ and nuclear PPARγ receptors, can also be the target of specific cannabinoids.

**Methods:**

We investigated the pro-neurogenic potential of the synthetic cannabigerol derivative, VCE-003.2, in striatal neurodegeneration by using adeno-associated viral expression of mutant huntingtin *in vivo* and mouse embryonic stem cell differentiation *in vitro*.

**Results:**

Oral administration of VCE-003.2 protected striatal medium spiny neurons from mutant huntingtin-induced damage, attenuated neuroinflammation and improved motor performance. VCE-003.2 bioavailability was characterized and the potential undesired side effects were evaluated by analyzing hepatotoxicity after chronic treatment. VCE-003.2 promoted subventricular zone progenitor mobilization, increased doublecortin-positive migrating neuroblasts towards the injured area, and enhanced effective neurogenesis. Moreover, we demonstrated the proneurogenic activity of VCE-003.2 in embryonic stem cells. VCE-003.2 was able to increase neuroblast formation and striatal-like CTIP2-mediated neurogenesis.

**Conclusions:**

The cannabigerol derivative VCE-003.2 improves subventricular zone-derived neurogenesis in response to mutant huntingtin-induced neurodegeneration, and is neuroprotective by oral administration.

**Electronic supplementary material:**

The online version of this article (10.1186/s40035-019-0148-x) contains supplementary material, which is available to authorized users.

## Background

Huntington’s disease (HD) is a monogenic neurodegenerative disease produced by the expression of mutant huntingtin (htt) protein with expanded glutamine repeats in the N-terminal portion of the protein [1]. Mutant htt expression induces striatal atrophy and medium spiny neuron (MSN) death, which is responsible for the characteristic motor symptoms of the disease. The length of the polyglutamine tract expansion in the htt gene, higher than 40 repeats, is closely associated with disease onset. Hence, although HD neurodegeneration occurs in adult brain, mutant htt expression is also known to interfere with normal neurodevelopment at several levels. Mutant huntingtin interferes with the activity of various transcription factors (GSX2+, ASCL1+, ISLT1+, NKX2.1) that are essential in controlling cortical and striatal neurogenesis [2, 3]. Among others, mutant huntingtin decreases NeuroD1 activity, a transcription factor that acts as a major confluence node of different neurodevelopmental gene pathways [3]. Mutant huntingtin also alters the mode of division of subventricular zone (SVZ) progenitors by influencing mitotic spindle orientation, that in turn controls asymmetric cell division and neurogenesis [4]. Moreover, mutant huntingtin expression interferes with projection neuron migration as normal huntingtin regulates Rab11-mediated N-cadherin trafficking [5].

Certain cannabinoids, the main active compounds of *Cannabis sativa*, and their endogenous counterparts 2-arachidonoylglycerol and anandamide exert symptomatic relief in various neurodegenerative and neuroinflammatory conditions [6]. Neuroprotection in mouse HD models as induced by Δ^9^-tetrahydrocannabinol (THC), the most abundant bioactive compound of *Cannabis sativa*, occurs at least in part in a cell-autonomous manner via the CB_1_ receptor [7]. Unfortunately, considering that CB_1_ receptor levels notably diminish at early stages of HD [8–10], and that CB_1_ receptor agonists produce undesired psychoactive effects [11], the development of clinical treatments based on CB_1_-receptor acting cannabinoids constitutes an extremely complicated task. Hence, investigating the potential use of cannabinoids other than THC, with different pharmacological profiles, constitutes an interesting research area for the development of new candidate molecules with reduced unwanted actions. Cannabigerol (CBG) is a non-psychoactive cannabinoid that has been tested as a candidate molecule for pharmacological therapies in HD experimental models [12]. CBG via the nuclear receptor peroxisome proliferator-activated receptor-γ (PPARγ) alleviates motor symptoms, neuroinflammation and neurodegeneration in murine models of HD based on either striatal neurotoxicity (3-nitropropionic acid injection model) or transgenic expression of human mutant huntingtin exon 1 (R6/2 model) [12]. Of interest, thiazolidinedione-induced PPARγ activation is neuroprotective against mutant huntingtin-induced cell death and reduce huntingtin aggregates in the brain [13]. In order to improve the pharmacological profile of CBG, chemical modifications were introduced, leading to the CBG quinone derivatives VCE-003 and VCE-003.2 [14, 15]. VCE-003.2 ([2-(3,7-dimethyl-octa-2, 6-dienyl)-6-ethylamino-3-hydroxy-5-pentyl-(1,4) benzoquinone]), an aminoquinone derivative of CBG, improves the pharmacological profile of VCE-003 by eliminating the characteristic side effects of potent PPARγ activators. Fundamentally, VCE-003.2 is neuroprotective in the quinolinic acid-administration model of HD and increases neural progenitor survival [14]. In the present study, we evaluated the neuroprotective efficacy of oral VCE-003.2 administration. Noteworthy, oral VCE-003.2 was neuroprotective against mutant huntingtin-induced damage, and also improved SVZ-derived neurogenesis. Overall, these findings provide further support to the neuroprotective and anti-inflammatory activities of VCE-003.2, and suggests its potential as a disease-modifying agent, considering its ability to improve the endogenous neurogenic response.

## Methods

### Cell culture and reagents

All reagents, unless indicated, were from Sigma-Aldrich (St. Louis, MO, USA). The synthesis and structure of VCE-003.2 were previously described [14, 15]. Mouse embryonic teratocarcinoma P19 were cultured in high-glucose DMEM (Invitrogen) supplemented with 10% fetal bovine serum and 1% penicillin/streptomycin. P19 cells were plated in 24-well plates and transfected with Lipofectamine 2000 reagent according to the manufacture’s protocol (Invitrogen). Twenty-four hours post transfection, cells were collected for RNA extraction or luciferase assay determinations. P19 neurosphere formation assay was performed using dissociated P19 cells cultured in high-glucose DMEM (Invitrogen) supplemented with 5% of fetal bovine serum and 0. 5 μM retinoic acid. Twenty-four hours later the mean neurosphere size was quantified. Luciferase construct with the MAR sequence A4 of the Ctip2 promoter was kindly provided (R. Grosschedl, Max-Planck-Institute of Immunobiology and Epigenetics, Freiburg, Germany).

### Mouse embryonic stem cell differentiation

R1 mouse embryonic stem (mES) cell line was grown on gelatin 0.1%, in Knockout DMEM supplemented with 20% Knockout Serum Replacement, 1000 U ml-1 of LIF, 0.1 mM of non-essential amino acids, 2 mM ultraglutamine, 50 U ml-1 of penicillin and streptomycin and 0.1 mM of 2-mercaptoethanol. Cells were then differentiated at low density in Defined Default Medium (DDM) containing DMEM/F12 + GlutaMAX supplemented with N2 supplement (1×), 5 mM of Hepes Buffer, 0.1 mM of non-essential amino acids, 1 mM of sodium pyruvate, 2.5 mg ml-1 of AlbuMax-I, 30 mM of D-Glucose, 0.1 mM of non-essential amino acids, 0.1 mM of 2-mercaptoethanol, 50 U ml-1 of penicillin and streptomycin for 12 days. The cells were then plated on polylysine/laminin-coated dishes in 50% DDM and 50% Neurobasal/B27 containing Neurobasal supplemented with B27 (1×), 2 mM of Ultraglutamine and 50 U ml-1 of penicillin and streptomycin for 9 days.

### Luciferase transcriptional assays

To study CTIP2 transcriptional activity P19 cells were seeded in 24-well plates and transiently co-transfected with the luciferase reporter vector MARS-A4-luc [16] using Roti-Fect (Carl Roth, Karlsruhe, Germany) following the manufacturer’s instructions. To correct for transfection efficacy, 100 ng *Renilla* luciferase (pRL-CMV) was also cotransfected. After stimulation, the luciferase activities were quantified using Dual-Luciferase Assay (Promega, Madison, WI, USA).

### Mutant huntingtin-induced neurodegeneration

**Fig. 1:**
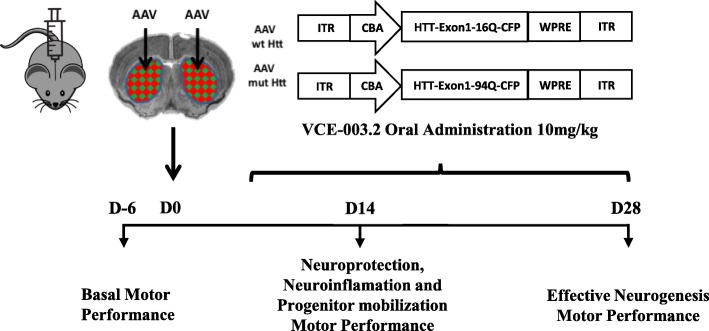
Experimental design for pharmacological manipulation and characterization of in vivo model of Huntington’s disease based on adeno-associated viral expression of mutant huntingtin exon1

Male C57BL/6 N mice (10 weeks old) were housed under standard conditions (12-h light/dark cycle) in groups with access to food and water *ad libitum*. All experiments were performed in accordance with European Union guidelines and approved by the Animal Research Ethics Committee of Complutense and Córdoba University. Procedures were designed to minimize the number of animals used and their suffering. Constructs expressing CFP-tagged human huntingtin exon 1 harboring a pathogenic polyQ tract of 94 CAG repeats or a normal, non-pathogenic polyQ tract of 16 CAG repeats (kindly provided by Dr. José J. Lucas, Severo Ochoa Molecular Biology Center, Madrid, Spain) were employed using an AAV1/AAV2 mixed serotype, generated by polyethyleneimine transfection of HEK293T cells and subsequent purification in iodixanol gradient. Vectors were injected stereotactically (in 3 μl PBS) into the dorsal striatum in a bilateral manner at coordinates (mm to bregma): antero-posterior + 0.5, lateral ±2.5, dorso-ventral − 3.5 as previously described [17]. VCE-003.2 (10 mg/kg) or vehicle (sesame oil) were orally administered once daily (Fig. 1), and for neurogenesis experiments BrdU (100 mg/kg) was administered i.p. twice daily on the first week after viral injection. RotaRod test was conducted prior to drug injections to avoid acute effects of the compounds under investigation. RotaRod started at 4 rpm, with an acceleration rate of 6 rpm/min until either maximum speed is reached or the mouse falls from the apparatus. Maximum time assay was 10 min. Basal RotaRod performance was determined 6 days prior to striatal injury in 3 consecutive days with 3 trials/day (30 min of rest between each trial), and groups with equivalent motor function were assigned. RotaRod test was performed during 3 consecutive days prior to sacrifice and plasma was obtained for peripheral biomarker analyses. All experiments included a minimum of 6 mice per condition. The precise number of animals analyzed in each experiment is indicated in the corresponding figure legends.

### Gene expression

Total RNA was isolated from striate using the Qiagen RNeasy Lipid kit (Qiagen, Germany). Total RNA (1 μg) was retrotranscribed using the iScript cDNA Synthesis Kit and the cDNA analyzed by real-time PCR using the iQTM SYBR Green Supermix and a CFX96 Real-time PCR Detection System (Bio-Rad; Hercules, CA, USA). The HPRT gene was used to standardize mRNA expression in each sample. Gene expression was quantified using the 2^-ΔΔCt^ method and the percentage of relative expression against controls (untreated cells or mice) was represented. The primers used in this study were: IL-6; forward: 5’GAACAACGATGATGCACTTGC3’; reverse: 5’TCCAGGTAGCTATGGTACTCC3’; TNFα; forward: 5’AGAGGCACTCCC CCAAAAGA-3; reverse: CGATCACCCCGAAGTTCCCATT.

### Pharmacokinetics

Six-week old male Sprague Dawley rats (Janvier Labs, France) were located in the rodent area of Eurofins ADME Bioanalyses (Vergèze, France) and housed under standard conditions (12-h light/dark cycle) in groups with access to food and water *ad libitum*. Each animal was identified by an ear tag and examined for general health and welfare. Process, treatment and euthanasia were conducted according to the current procedures in use at Eurofins ADME Bioanalyses. Animals were treated with a single intravenous bolus of VCE-003.2 (10 mg/Kg in DMSO) or with oral VCE-003.2 (50 mg/Kg in sesame oil). At the prescribed times, blood (0.5 ml) was collected using a capillary tube and plasma frozen at − 20 °C until analysis. After 24 h, the animals were anesthetized and perfused with 20 mL of saline solution directly into the heart to extract the maximal blood sample from the brain. Plasma samples (100 μl) were mixed with 300 μl of acetonitrile and after protein precipitation, analysis of VCE-003.2 content was performed using LC-MS/MS (3 animals per each time point). For brain samples, the tissues were homogenized with an Ultra-turrax® using UHQ water (1/1, *w*/w). 100 μl of each homogenate was mixed with 300 μl of acetonitrile and the mixture centrifuged for 5 min at 25,000 g. Brain homogenate supernatants were directly measured by LC-MS/MS. Previous studies have shown that there are not significant differences between mice and rats in the main pharmacokinetic parameters of different cannabinoids, including CBG [18]. As the dose used here was different between i.v. and oral routes of administration, bioavailability was calculated taking into account the dose as follows: [AUCt (oral)/dose (oral)]/[AUCt(i.v.)/dose(i.v.)].

### Proteome array

Plasma samples from wild-type and mutant huntingtin-expressing C57BL/6 N mice were pooled (*n* = 6 animals per group) and analyzed for cytokine and adipokine expression. The Proteome Profiler Mouse XL Cytokine Array and the Proteome Profiler Mouse Adipokine Array (R&D Systems) were used according to the manufacturer’s protocols to obtain protein expression profiles using 100 μl plasma samples. Spot density was determined using Quick Spots image analysis software (R&D Systems).

### Immunofluorescence and confocal microscopy

Free floating coronal brain slices (30 μm) were processed as previously described [17]. In brief, after blocking with 10% goat serum, brain sections were incubated with the indicated primary antibodies followed by secondary antibody incubation (2 h at room temperature). The appropriate mouse, rat and rabbit highly cross-adsorbed AlexaFluor 488, AlexaFluor 594 and AlexaFluor 647 secondary antibodies (1:500; Molecular Probes, Leyden, The Netherlands) were used. Samples were subsequently incubated with DAPI (1:5000, Roche) for 10 min, washed with PBS and mounted in Mowiol. All immunofluorescence data were obtained in a blinded manner by independent observers in a minimum of 6 correlative slices, from 1-in-10 series located between − 0.4 to + 1.6 mm to bregma. The lateral SVZ zone was delineated by using DAPI-counterstained cell nuclei, and analyses were performed in the upper dorsal tier, in which positively-labelled cells and immunoreactivity were quantified not beyond 100 μm of the SVZ. Confocal fluorescence images were acquired by using LAS-X software (Wetzlar, Germany) and SP8 microscope with 2 passes by Kalman filter, a 1024X1024 collection box, and pinhole AU 1. Double-labelled positive cells (GFAP/Ki-67 and BrdU/NeuN cells) were counted at a magnification of 40×. Cells were quantified within a 20 × 20 μm counting frame, which randomly sampled within a 122.8 × 68.9 μm counting grid. Cells that contacted the lateral or upper exclusion plane were excluded. The total number of cells counted was divided by the number of sampled counting frames and multiplied by its size to obtain the density of positive cells. Co-localization was confirmed by orthogonal projection of 18 z-stack files (1-μm each), and data were expressed as cells/mm^2^. Neurodegeneration and glial activation was determined by dopamine- and cAMP-regulated phosphoprotein of 32 kDa mouse anti-DARPP32 (1:500 BD Transduction Laboratories, Lexington, KY), rabbit anti-Iba-1 (1:500 Wako Pure Chemical, Osaka, Japan) and mouse anti-GFAP-Cy3 (1:500 Sigma, St. Louis, MO) immunostaining, and quantified with Image-J software designed by the National Institutes of Health (NIH; Bethesda, MD, USA). Neurogenesis and SVZ-progenitor mobilization were characterized by immunofluorescence with rat anti-CTIP2 antibodies (1:500 Abcam, Cambridge, United Kingdom). Rabbit anti-Doublecortin (1:1000 from Abcam), rat anti-BrdU (1:250 from Abcam) and mouse anti-NeuN (1:500 from Chemicon) antibodies, or rabbit anti-GFAP and rabbit anti-Ki67 (1:400 from Invitrogen and 1:500 from ThermoScientific).

### Statistical analysis

*In vitro* data are expressed as mean ± S.D. and *in vivo* results are represented as mean ± SEM. Data were subjected to Kolmogorov-Smirnov normality test and then, differences were analyzed by one-way ANOVA followed by Tukey post hoc test. *P* < 0.05 was considered significant. Statistical analysis was performed using GraphPad Prism version 5.01 and is shown in figure legends. Images were analyzed and quantified using the ImageJ.

## Results

### VCE-003.2 exerts a pro-neurogenic effect *in vitro*

**Fig. 2:**
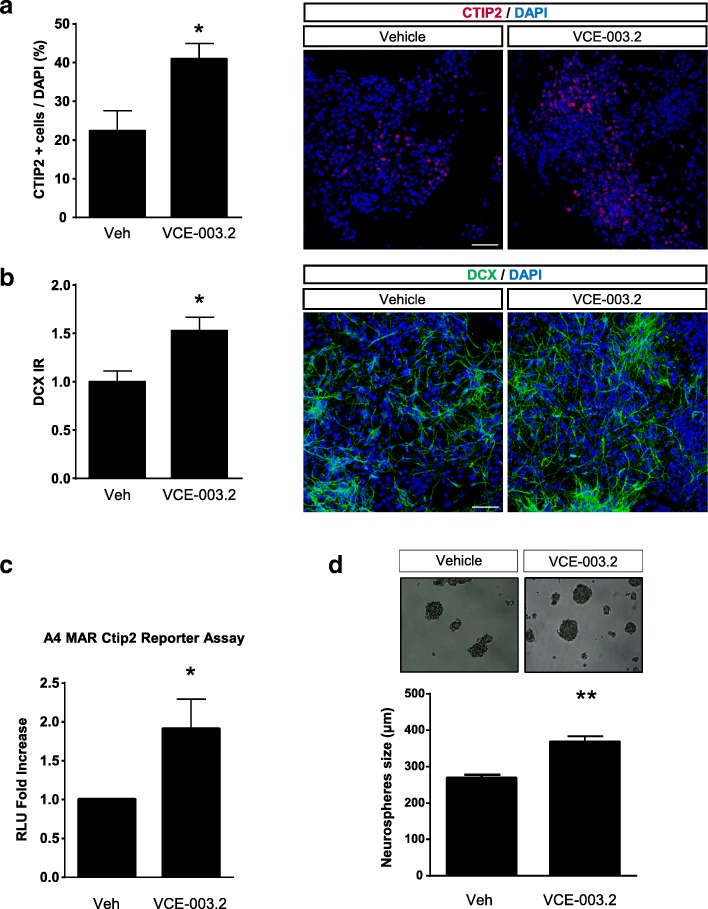
VCE-003.2 exerts a pro-neurogenic effect in vitro. **a-b** Mouse embryonic stem cells (R1 line) were treated with Vehicle, VCE-003.2 (500 nM) during neural differentiation for 21 days. Representative immunofluorescence images and quantification of CTIP2- and doublecortin-positive cells is shown (*n* = 3). **c** Luciferase reporter assay of the A4-MARS sequence of the CTIP2 locus was performed 24 h after P19 cell transfection under neuronal differentiation conditions and subject to pharmacological regulation as above. **d** P19 neurosphere formation assay was performed by culturing the cells for 24 h in the presence of Vehicle or VCE-003.2. Statistics: Unpaired t-test vs Vehicle **a** * *p* < 0.05; t = 2.82; df = 4; 95% Confidence interval (CI) = 0.3149 to 36.85; R^2^ = 0.66. **b** * *p* < 0.05; t = 2.91; df = 4; 95% CI = 0.02 to 1.03; R^2^ = 0.67. **c** * *p* < 0.05; t = 2.87; df = 5; 95% CI = 0.09 to 1.72; R^2^ = 0.62. **d** ** < 0.01 t = 5.91; df = 362; 95% CI = 66.20 to 132.20; R2 = 0.08. Scale bar, 50 μm

To investigate the pro-neurogenic potential of VCE-003.2 we analyzed its influence in ES-neuronal differentiation. The R1 line of mouse ES cells was treated with VCE-003.2 during neural differentiation for 21 days and we assessed CTIP2-positive striatal MSN differentiation [19]. Immunofluorescence quantification revealed that VCE-003.2 increased the number of CTIP2-positive cells as well as doublecortin immunoreactivity (Fig. 2a-b). Next, using neuralized mouse embryonic teratocarcinoma P19 cells, we performed CTIP2 transcriptional activity assays by transfecting a luciferase reporter construct (A4-Mar) corresponding to one of the regulatory MARS sequences of the CTIP2 promoter. VCE-003.2 promoted neuronal-like differentiation as revealed by CTIP2 reporter activation (Fig. 2c). Furthermore, using a P19 neurosphere formation assay, VCE-003.2 generated larger neurospheres than vehicle-treated cells (367.90 ± 15.20 μm and 268.70 ± 9.20 μm, respectively; Fig. 2d). These results support a pro-neurogenic action of VCE-003.2 on neural stem cell differentiation.

### Pharmacokinetics and bioavailability of VCE-003.2

As VCE-003.2 was previously shown to exert a neuroprotective action in preclinical HD models, and considering its pro-neurogenic profile, we evaluated the pathophysiological impact and potential therapeutic value of VCE-003.2 after oral administration. Pharmacokinetic analyses were performed in rats after oral administration of VCE-003.2 (50 mg/kg) dissolved in sesame oil. Plasma VCE-003.2 levels peaked at 8 h (Tmax) and then slowly declined to basal levels. Oral VCE-003.2 resulted in 13.8% bioavailability and after 24 h the concentration of the compound in the brain was similar upon oral or i.v. treatment (Table 1). We also investigated some critical ADME/Tox parameters. By using human liver microsomes we found that VCE-003.2 undergoes a rapid intrinsic clearance *in vitro,* which was similar to the parental compound CBG (Additional file 1). Moreover, VCE-003.2 did not inhibit significantly the activity of relevant cytochrome P450 isoforms (Additional file 2). We also determined the potential hepatotoxicity of chronic VCE-003.2 administration by hematoxylin-eosin staining. VCE-003.2- and vehicle-treated mouse-derived livers did not show any evidence of fibrosis, steatosis, vacuolization, ballooning or inflammation (data not shown). In addition, VCE-003.2 administration did not inhibit hERG channel activity, suggesting a potential lack of cardiotoxicity (Additional file 3), and it was not genotoxic as assessed by AMES assays (Additional file 4). We also determined the impact of VCE-003.2 administration on peripheral biomarkers by quantifying the levels of cytokines and other soluble mediators. Viral infection lead to changes in various neuroinflammation biomarkers, notably C-reactive protein and pentraxins 2, 3 were increased by htt94Q and these changes were reverted by VCE-003.2 treatment (Additional file 5). These data indicate that mutant huntingtin induces a process of neuroinflammation that results in the release of soluble factors that can be quantified in plasma and normalized by VCE-003.2 oral administration.

**Table 1:** Pharmacokinetic parameters of VCE-003.2. Pharmacokinetic parameters of VCE-003.2 in plasma following a single intravenous (i.v.) (10 mg/kg) and oral (50 mg/kg) dose in Sprague Dawley rats

Compound	Route	Cmax (ng/mL)	Tmax (h)	AUCt ng/mL*h)	Bioavailability	Brain Concentration (24 h) (ng/mL)
VCE-003.2	IV	83160	0.08	475094.96		93.7 ± 37.7
VCE-003.2	ORAL	20266.67	8	327154.14	13.77%	86.8 ± 34.0

### Oral administration of VCE-003.2 attenuates neuroinflammation and is neuroprotective in a viral model of mutant huntingtin expression

**Fig. 3:**
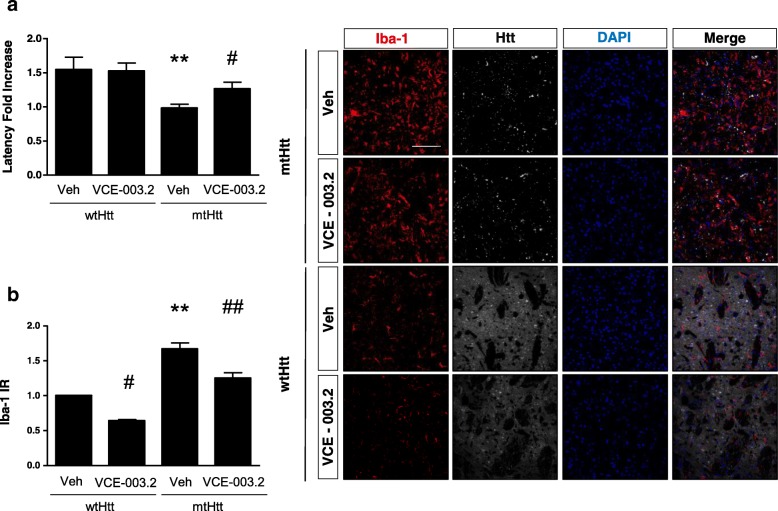
Oral administration of VCE-003.2 attenuates microglial activation and motor impairment induced by mutant-huntingtin expression. Wild type C57Bl/6 N mice were injected bilaterally with mutant huntingtin expressing AAV-htt94Q and AAV-htt16Q as control. VCE-003.2 or vehicle were administered orally daily (10 mg/kg) and mice analyzed at 2 after lesion. **a** Motor function was assessed in the RotaRod test and mean latency to fold quantified. **b** Representative confocal microscopy images of huntingtin-CFP and immunoreactivity with an antibody for microglia (Iba1). Iba1 immunoreactivity was quantified in the indicated experimental groups. AAV-htt16Q Vehicle and VCE-003.2 (*n* = 3 and 6, respectively), AAV-htt94Q Vehicle and VCE-003.2 (*n* = 3 and 5, respectively). Statistics: One-way ANOVA. **a** F = 5.94; R^2^ = 0.29. **p < 0.01; q = 4.88 AAV-htt16Q Veh vs. Mut-htt94q Veh and #*p* < 0.05; q = 3.90 vs AAV-htt94Q Veh vs. AAV-htt94Q VCE-003.2. **b** F = 5.87; R^2^ = 0.91. #*p* < 0.05; q = 5.32 vs AAV-htt16Q VCE-003.2 vs. AAV-htt94Q Veh and ##*p* < 0.01; q = 3.49 vs AAV-htt94Q VCE-003.2 vs. AAV-htt94Q Veh. **p < 0.01; q = 9.30 AAV-htt94Q Veh vs. AAV-htt16Q Veh. Scale bar, 100 μm

**Fig. 4:**
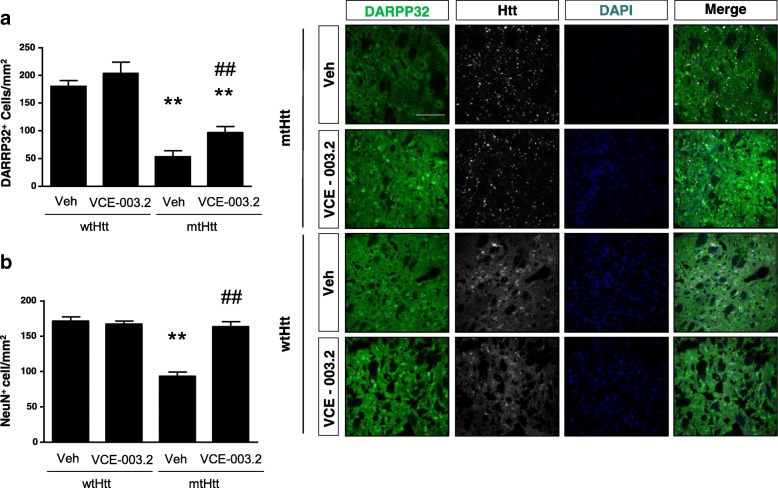
Oral administration of VCE-003.2 is neuroprotective from mutant-huntingtin-induced neurodegeneration. Wild type mice were injected bilaterally with mutant huntingtin expressing AAV-htt94Q (mtHtt) and AAV-htt16Q (wtHtt) as control. VCE-003.2 or vehicle were administered orally daily (10 mg/kg) and mice analyzed 30 days after lesion. Confocal microscopy characterization of huntingtin-CFP and using an antibody for the MSN marker DARPP32. Quantification of MSN survival after lesion for the indicated experimental groups. AAV-htt16Q (Vehicle and VCE-003.2, *n* = 5 each) and AAV-htt94Q (Vehicle and VCE-003.2, *n* = 7 each). Statistics: One-way ANOVA. **a** F = 31.56; R^2^ = 0.80. ##*p* < 0.01; q = 5.28 AAV-htt94Q VCE-003.2 vs. AAV-htt94Q Veh. **p < 0.01; q = 10.63 vs AAV-htt94Q Veh vs. AAV-htt94Q Veh vs. AAV-htt16Q Veh and **p < 0.01; q = 5.56 vs AAV-htt94Q VCE-003.2 vs. AAV-htt16Q Veh. **b** F = 27.15; R^2^ = 0.78. ##*p* < 0.01; q = 10.74 AAV-htt94Q VCE-003.2 vs. AAV-htt94Q Veh and **p < 0.01; q = 10.85 vs AAV-htt94Q Veh vs. AAV-htt16Q Veh. Scale bar, 100 μm

To evaluate the neuroprotective profile of oral VCE-003.2 administration, a viral model of HD was employed [20] in which wild-type mice were subjected to bilateral intrastriatal injection of adeno-associated virus (AAV) expressing either exon 1 of human pathogenic huntingtin with a polyQ tract of 94 CAG repeats, or a normal, non-pathogenic huntingtin with a polyQ tract of 16 CAG repeats (AAV-htt16Q and AAV-htt94Q, respectively), and treated with VCE-003.2 (10 mg/kg/day). Whereas expression of (exon1)-huntingtin-16Q during 14 days did not affect motor coordination, mutant huntingtin-94Q decreased RotaRod latency to fall (Fig. 3a). Motor impairment in htt94Q mice was accompanied by profound activation of microglial cells, as evidenced by Iba1 confocal immunofluorescence, in the area surrounding the infection site (Fig. 3b). Mice treated with VCE-003.2 performed better in the RotaRod test than their vehicle-treated AAV-htt94Q counterparts, and showed reduced microglial activation (Fig. 3a-b). Furthermore, VCE-003.2 administration prevented htt94Q-induced MSN degeneration as evidenced by DARPP-32 and NeuN immunofluorescence (Fig. 4a-b). These results are in line with previous findings on the neuroprotective ability of VCE-003.2 in toxin-based models of neurodegeneration [14, 21] and expand its potential clinical profile since oral administration was as effective as i.p. delivery. Likewise, oral VCE-003.2 administration was also neuroprotective and anti-inflammatory in the 3-NP model of striatal neurodegeneration (Additional file 6).

### Oral administration of VCE-003.2 promotes striatal neurogenesis

**Fig. 5:**
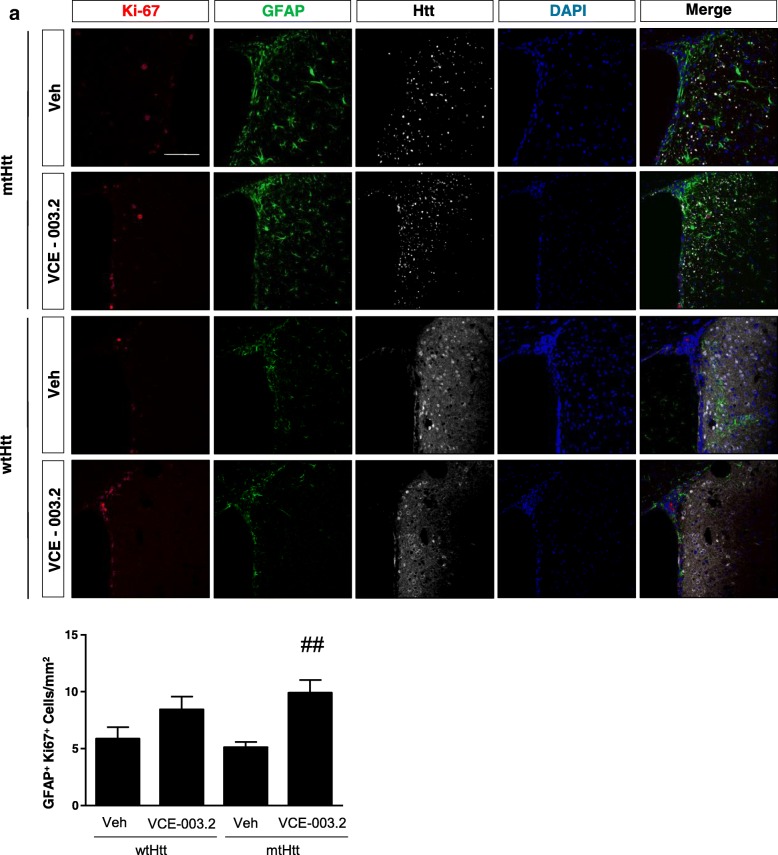
Subventricular zone neural progenitor mobilization is increased by oral administration of VCE-003.2. Mice were analyzed 4 weeks after lesion induced by AAV-htt16Q and AAV-htt94Q bilateral injection and daily treatment with vehicle or VCE-003.2 (10 mg/kg). Confocal microscopy characterization of the SVZ was performed with GFAP, htt and Ki-67-specific antibodies in the indicated experimental groups. Proliferating SVZ-neural stem cells were quantified based on GFAP and Ki-67 immunofluorescence colocalization. AAV-htt16Q (Veh and VCE-003.2, *n* = 7 and 6, respectively) and AAV-htt94Q (Veh and VCE-003.2, *n* = 7 and 8, respectively). Statistics: One-way ANOVA. F = 5.78; R^2^ = 0.42. ##*p* < 0.01; q = 5.29 AAV-htt94Q VCE-003.2 vs. AAV-htt94Q Veh. Scale bar, 100 μm

**Fig. 6:**
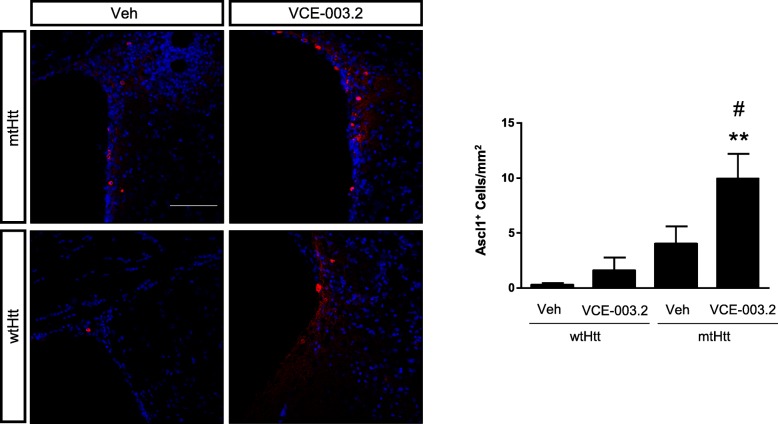
Subventricular zone neural progenitor mobilization is increased by oral administration of VCE-003.2. Mice were analyzed 4 weeks after lesion induced by AAV-htt16Q and AAV-htt94Q bilateral injection and daily treatment with vehicle or VCE-003.2 (10 mg/kg). Confocal microscopy characterization of the SVZ was performed with Ascl1-specific antibody in the indicated experimental groups. Quantification of transit amplifying progenitors labelled with Ascl1. AAV-htt-16Q (Vehicle and VCE-003.2, *n* = 5 each) and AAV-htt94Q (Vehicle and VCE-003.2, *n* = 5 each). Statistics: One-way ANOVA. F = 10.12; R^2^ = 0.65. ##*p* < 0.01; q = 4.25 AAV-htt94Q VCE-003.2 vs. AAV-htt94Q Veh and **p < 0.01; q = 7.12 AAV-htt94Q VCE-003.2 vs. AAV-htt16Q Veh. Scale bar, 100 μm

**Fig. 7:**
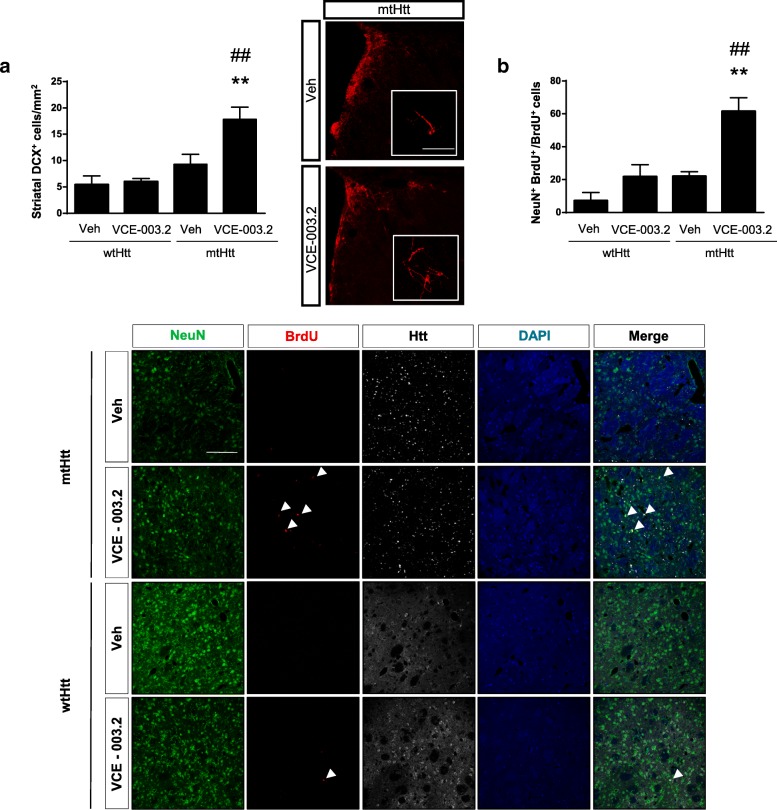
Oral administration of VCE-003.2 exerts a pro-neurogenic action. Mice were analyzed 4 weeks after lesion induced by AAV-htt16Q and AAV-htt94Q bilateral injection and administered daily with vehicle or VCE-003.2 (10 mg/kg). **a** Confocal microscopy characterization of migrating neuroblasts identified with doublecortin antibody and quantification in the striatum of the indicated mice groups. **b** Effective neurogenesis was determined by quantification of double positive cells labelled with BrdU and NeuN antibodies. AAV-htt16Q Vehicle and VCE-003.2 (*n* = 7 and 5, respectively); AAV-htt94Q Vehicle and VCE-003.2 (*n* = 9 and 11, respectively). Statistics: One-way ANOVA. **a** F = 14.43; R^2^ = 0.69. ##*p* < 0.01; q = 6.95 AAV-htt94Q VCE-003.2 vs. AAV-htt94Q Veh and **p < 0.01; q = 7.59 AAV-htt94Q VCE-003.2 vs. AAV-htt16Q Veh. **b** F = 13.99; R^2^ = 0.59. ##p < 0.01; q = 6.50 AAV-htt94Q VCE-003.2 vs. AAV-htt94Q Veh and **p < 0.01; q = 7.59 AAV-htt94Q VCE-003.2 vs. AAV-htt16Q Veh. Scale bar, 100 μm

The neuroprotective action of oral VCE-003.2 and its pro-neurogenic impact *in vitro* prompted us to investigate if this compound could contribute to striatal neurorepair *in vivo* by promoting SVZ-derived neurogenesis. We analyzed the SVZ neural stem cell population, identified as double-labelled GFAP/Ki-67 cells, which represents the radial glia type B cell compartment. VCE-003.2 increased the number of proliferating GFAP-positive cells in AAV-htt94Q-injected mice, indicating its positive impact on NSC mobilization (Fig. 5). Next, SVZ-derived neural progenitors were identified by immunofluorescence against Ascl1 (a transcription factor characteristic of the transit amplifying progenitor subpopulation). Mutant huntingtin expression induced an increase in Ascl1^+^ cell number, and VCE-003.2 administration further promoted Ascl1-positive cell expansion in AAV-htt94Q-mice (Fig. 6). To determine the impact of VCE-003.2 on striatal neurogenesis we next evaluated doublecortin-positive neuroblasts after 30 days of AAV-mediated huntingtin expression and daily VCE-003.2 administration. AAV-htt94Q-induced injury resulted in a slight increase in neuroblast formation (Fig. 7a) and effective neurogenesis (BrdU^+^NeuN^+^ cells) (Fig. 7b) as compared to AAV-htt16Q. Oral administration of VCE-003.2 was effective in promoting neurogenesis in AAV-htt16Q- and AAV-htt94Q-treated mice, both determined as increased doublecortin-expressing cells and double-positive NeuN/BrdU neurons (Fig. 7a-b). Hence, oral VCE-003.2 administration is able to restore striatal neurogenesis affected by mutant huntingtin expression.

## Discussion

VCE-003.2 is an aminoquinone derivative of CBG that has been designated by the FDA as an orphan drug for HD treatment (www.accessdata.fda.gov/scripts/opdlisting/oopd/detailedIndex.cfm?cfgridkey=623717). In the present study, we demonstrate the pro-neurogenic activity of this cannabinoid derivative in a preclinical model of HD that adds to its neuroprotective activity. Oral administration of VCE-003.2 exerted an anti-inflammatory and neuroprotective action in a HD experimental model. In addition, VCE-003.2 was able to augment mutant huntingtin-induced SVZ-derived neurogenesis, thus favoring neural stem cell mobilization (double-labelled GFAP^+^/Ki-67^+^ and Ascl1^+^ cells), neuronal differentiation (doublecortin^+^ cells) and effective neurogenesis (double-labelled Brdu^+^/NeuN^+^ cells). In agreement with previous studies, mutant huntingtin expression induced a pro-neurogenic response that was evident by the trend towards increased Ascl1^+^, Dcx^+^ and double-labelled BrdU^+^/NeuN^+^ cells. The molecular signals and mechanisms underlying injury-induced neurogenesis are diverse, and numerous therapeutic approaches aim to promote this endogenous process strategy [22]. Hence, the results obtained in this study support that striatal neurogenesis is improved by oral VCE-003.2 administration. This new mechanism of action of VCE-003.2 is relevant when considering its potential clinical applications, as it suggests that this molecule could constitute a disease-modifying drug rather than merely symptom-palliative. It is worth noting that the preclinical model of HD employed in this study is based on the expression of exon 1 of either mutant huntingtin or normal, non-pathogenic huntingtin. The validity of this approach for mechanistic and pharmacological investigations is demonstrated by the high number of studies using it [23]. However, improved animal models, for example based on the expression of full-length huntingtin, are likely to provide novel insights of additional aspects of HD.

The use of plant derived-cannabinoid ligands has been proposed to exert symptomatic relief of motor symptoms in neurodegenerative diseases, including HD-associated dystonia and chorea, levodopa-induced dyskinesia and tremor in Parkinson’s disease, and multiple sclerosis-associated spasticity [6, 24]. Despite the great interest in the therapeutic potential of cannabinoids in neurodegenerative diseases, the undesired side-effects of CB_1_ receptor agonists significantly limit their use in clinical practice. In addition, during HD progression presynaptic CB_1_ receptor levels decline very early, even at presymptomatic stages [7, 9, 10]. Hence, despite the fact that corticostriatal projections preserve CB_1_ receptor expression [17], the use of CB_1_ agonists alone may have a limited therapeutic window, and the development of new cannabinoid-derived molecules acting via different signaling mechanisms and with reduced undesired side-effects constitutes an attractive strategy to solve these limitations.

PPARγ receptors are required for appropriate neurogenesis as it controls neural progenitor cell proliferation and differentiation [25]. Noteworthy, the positive effect of PPARγ in neurogenesis, requires fine-tune regulation, as either its ablation or overactivation may hamper neuronal differentiation [26]. Under pathological conditions, for instance under an inflammatory insult, PPARγ activation can restore neurogenesis and cognitive impairment [27]. Similarly, PPARγ activation can restore neurogenesis in the OH-DA model of Parkinson’s disease [28] and prevent amyloid-beta-induced cell death of human neural stem cells [29]. In HD models, the administration of PPARγ agonists protects from striatal neurodegeneration, attenuates neuroinflammation and decreases oxidative damage [30–32], supporting PPARγ as a valid target for the management of HD.

CBD is the most widely investigated phytocannabinoid devoid of psychotomimetic actions, and whereas its precise molecular mechanism of action remains the subject of research, some CBD actions, including its protective effect in counteracting amyloid-induced inflammation and neurogenesis deficits, are mediated by PPARγ receptors [33]. Hence, CBD administration can restore hippocampal neurogenesis deficits induced by different nervous system insults such as amyloid-beta pathology, chronic unpredictable stress and aging [34]. Other plant-derived cannabinoids such as CBG and tetrahydrocannabinolic acid regulate PPARγ activity [15, 35] and, hence, constitute interesting template structures for the development of new molecules with improved selectivity, reduced side-effects and better pharmacokinetic profile. Different strategies have been applied to improve CBD pharmacological properties. Fluorinated derivatives or quinone modifications have successfully generated new cannabinoid molecules with better translational perspectives (e.g., HUF-101, VCE-004). Thus, HUF-101 possesses improved anxiolytic, antidepressant and antipsychotic properties compared to the original CBD molecule [36]. Regarding CBG, its aminoquinone derivative VCE-003.2 has demonstrated efficacy as a neuroprotective molecule in HD [14], Parkinson’s disease [21] and amyotrophic lateral sclerosis models [37]. Noteworthy, the VCE-003.2 compound is devoid of the side effects seen with full PPARγ-agonists and, hence, does not interfere with osteoblast differentiation and is less adipogenic [14].

Cell replacement therapy has demonstrated efficacy in HD experimental models and in preliminary studies in patients [38]. Exogenous cell grafts of either fetal tissue or stem-cell derived neurons are able to survive in the striatum, as well as integrate and ameliorate motor symptoms and survival, however they face various challenges that impede their development for disease-modifying strategy. Considering that in HD postmortem tissue an increased SVZ cell proliferation and neurogenesis is evident [39], promoting endogenous SVZ-derived striatal neurogenesis constitutes an alternative approach of interest. Promoting SVZ neurogenesis by gene therapy-mediated delivery of BDNF and noggin successfully delayed progression of the disease in the R6/2 mouse model [40, 41], and the same approach induced neurogenesis in adult squirrel monkeys [40]. These findings evidence that, even if endogenous newly-born neurons express mutant huntingtin, neurogenesis is effective and able to counteract, at least in part, neurodegeneration-evoked symptoms. In this scenario, pharmacological promotion of adult neurogenesis by cannabinoids [42, 43] may constitute a plausible alternative to gene therapy. In addition, cannabinoids could be used to promote ES-derived striatal neurogenesis *ex vivo* for mechanistic studies or cell replacement therapies.

In summary, our findings demonstrate that oral administration of VCE-003.2 exerts a neuroprotective action in a striatal neurodegeneration model that is accompanied by an improved endogenous neurogenic response. The capability of VCE-003.2 to increase effective neurogenesis suggests that this CBG derivative may possess the ability to act as a disease-modifying drug rather than only a symptomatic reliever.

## Conclusions

Oral administration of the cannabigerol derivative VCE-003.2 is neuroprotective and improves subventricular zone-derived neurogenesis in response to mutant huntingtin-induced neurodegeneration. These findings are relevant in the search for novel therapeutic strategies against Huntington’s disease progression, considering the lack of undesired side actions of this novel cannabinoid-derived molecule and good bioavailability upon oral administration.

## Additional files

Additional file 1:Microsomal Metabolic Stability. Pooled human liver microsomes (final protein concentration 0.5 mg/mL), 0.1 M phosphate buffer pH 7.4 and the test compounds (VCE-003.2, CBG, verapamil and dextromethorphan) were pre-incubated at 37 °C prior to the addition of 1 mM NADPH to initiate the reaction. The final incubation volume was 25 μL. Each compound was incubated for 0, 5, 15, 30 and 45 min. The control (minus NADPH) was incubated for 45 min only. The reactions were stopped by the addition of 50 μL methanol containing internal standard at the appropriate time points. The incubation plates were centrifuged at 2500 rpm for 20 min at 4 °C to precipitate the protein. Following protein precipitation, the sample supernatants were analyzed using LC-MS/MS. From a plot of ln peak area ratio (compound peak area/internal standard peak area) against time, the gradient of the line was determined. Subsequently, half-life and intrinsic clearance was calculated using the equations below: Elimination rate constant (k) = (− gradient). Half-life (t_1/2_) (min) = $\frac{0.693}{k}$. Intrinsic Clearance (CL_int_) (μL/min/mg protein) = $\frac{V\times 0.693}{t_{1/2}}$. where V=Incubation volume mL/mg microsomal protein. (PDF 373 kb)

Additional file 2: Cytochrome P450 Inhibition (IC50 Determination). **CYP1A Inhibition.** VCE-003.2 (0.1, 0.25, 1, 2.5, 10, 25 μM in DMSO; final DMSO concentration = 0.3%) was incubated with human liver microsomes (0.25 mg/mL) and NADPH (1 mM) in the presence of the probe substrate ethoxyresorufin (0.5 μM) for 5 min at 37 °C. The selective CYP1A inhibitor, alpha-naphthoflavone, was screened alongside the test compounds as a positive control. **CYP2B6 Inhibition.** VCE-003.2 (0.1, 0.25, 1, 2.5, 10, 25 μM in DMSO; final DMSO concentration = 0.3%) was incubated with human liver microsomes (0.1 mg/mL) and NADPH (1 mM) in the presence of the probe substrate bupropion (110 μM) for 5 min at 37 °C. The selective CYP2B6 inhibitor, ticlopidine, was screened alongside the test compounds as a positive control. **CYP2C8 Inhibition.** VCE-003.2 (0.1, 0.25, 1, 2.5, 10, 25 μM in DMSO; final DMSO concentration = 0.3%) was incubated with human liver microsomes (0.25 mg/mL) and NADPH (1 mM) in the presence of the probe substrate paclitaxel (7.5 μM) for 30 min at 37 °C. The selective CYP2C8 inhibitor, montelukast, was screened alongside the test compounds as a positive control. **CYP2C9 Inhibition.** VCE-003.2 (0.1, 0.25, 1, 2.5, 10, 25 μM in DMSO; final DMSO concentration = 0.3%) was incubated with human liver microsomes (1 mg/mL) and NADPH (1 mM) in the presence of the probe substrate tolbutamide (120 μM) for 60 min at 37 °C. The selective CYP2C9 inhibitor, sulphaphenazole, was screened alongside the test compounds as a positive control. **CYP2C19 Inhibition.** VCE-003.2 (0.1, 0.25, 1, 2.5, 10, 25 μM in DMSO; final DMSO concentration = 0.3%) was incubated with human liver microsomes (0.5 mg/mL) and NADPH (1 mM) in the presence of the probe substrate mephenytoin (25 μM) for 60 min at 37 °C. The selective CYP2C19 inhibitor, tranylcypromine, was screened alongside the test compounds as a positive control. **CYP2D6 Inhibition.** VCE-003.2 (0.1, 0.25, 1, 2.5, 10, 25 μM in DMSO; final DMSO concentration = 0.3%) was incubated with human liver microsomes (0.5 mg/mL) and NADPH (1 mM) in the presence of the probe substrate dextromethorphan (5 μM) for 5 min at 37 °C. The selective CYP2D6 inhibitor, quinidine, was screened alongside the test compounds as a positive control. **CYP3A4 Inhibition.** VCE-003.2 (0.1, 0.25, 1, 2.5, 10, 25 μM in DMSO; final DMSO concentration = 0.3%) was incubated with human liver microsomes (0.1 mg/mL) and NADPH (1 mM) in the presence of the probe substrate midazolam (2.5 μM) for 5 min at 37 °C. The selective CYP3A4 inhibitor, ketoconazole, was screened alongside the test compounds as a positive control. **CYP3A4 Inhibition.** VCE-003.2 (0.1, 0.25, 1, 2.5, 10, 25 μM in DMSO; final DMSO concentration = 0.3%) was incubated with human liver microsomes (0.5 mg/mL) and NADPH (1 mM) in the presence of the probe substrate testosterone (50 μM) for 5 min at 37 °C. The selective CYP3A4 inhibitor, ketoconazole, was screened alongside the test compounds as a positive control. For the CYP1A incubations, the reactions were terminated by methanol, and the formation of the metabolite, resorufin, was monitored by fluorescence (excitation wavelength = 535 nm, emission wavelength = 595 nm). For the CYP2B6, CYP2C9, CYP2C19, CYP2D6, and CYP3A4 incubations, the reactions were terminated by methanol. The samples were centrifuged, and the supernatants combined for the simultaneous analysis of 4-hydroxytolbutamide, 4-hydroxymephenytoin, dextrorphan, and 1-hydroxymidazolam by LC-MS/MS. Hydroxybupropion, 6α-hydroxypaclitaxel and 6ß-hydroxytestosterone were analyzed separately by LC-MS/MS. A decrease in the formation of the metabolites compared to vehicle control was used to calculate IC50 values. (PDF 323 kb)

Additional file 3: hERG Channel Inhibition (IC_50_ Determination). The experiments were performed on an IonWorks™ HT instrument (Molecular Devices Corporation), which automatically performs electrophysiology measurements in 48 single cells simultaneously in a specialized 384-well plate (PatchPlate™). The cells used were Chinese hamster ovary (CHO) cells stably transfected with hERG (cell-line obtained from Cytomyx, UK). A single-cell suspension was prepared in extracellular solution (Dulbecco’s phosphate buffered saline with calcium and magnesium pH 7–7.2) and aliquots added to each well of a PatchPlate™. Cells were positioned over a small hole at the bottom of each well by applying a vacuum beneath the plate to form an electrical seal. The resistance of each seal was measured via a common ground-electrode in the intracellular compartment and individual electrodes placed into each of the upper wells. Electrical access to the cell was achieved by circulating a perforating agent, amphotericin, underneath the PatchPlate™ and then measuring the pre-compound hERG current. An electrode is positioned in the extracellular compartment and a holding potential of − 80 mV applied for 15 s. The hERG channels were then activated by applying a depolarizing step to + 40 mV for 5 s and then clamped at − 50 mV for 4 s to elicit the hERG tail current, before returning to − 80 mV for 0.3 s. VCE-003.2 were added to the upper wells of the PatchPlate™. Solutions were prepared by diluting 10 mM DMSO solutions of the test compound into extracellular buffer such that the final concentrations tested are 0.008, 0.04, 0.2, 1, 5 and 25 μM (final DMSO concentration 0.25%). Quinidine, an established hERG inhibitor, was included as a positive control and buffer containing 0.25% DMSO was included as a negative control. Post-compound currents were then expressed as a percentage of pre-compound currents and plotted against concentration for each compound. Where concentration-dependent inhibition is observed, the data are fitted and IC_50_ values were calculated. (PDF 322 kb)

Additional file 4: AMES Data Summary. AMES Experimental Procedure. Approximately ten million bacteria are exposed in triplicate to VCE-003-.2 (7.8, 15.6, 31.3, 62.5, 125 and 250 μg/ml), a negative control (vehicle) and a positive control for 90 min in medium containing a low concentration of histidine (sufficient for about 2 doublings). The cultures were then diluted into indicator medium lacking histidine and dispensed into 48 wells of a 384 well plate (micro-plate format, MPF). The plate was incubated for 48 h at 37 °C, and the number of wells showing growth were counted and compared to the vehicle control. An increase in the number of colonies of at least two-fold over baseline (mean + SD of the vehicle control) and a dose response indicates a positive response. An unpaired, one-sided Student’s T-test was used to identify conditions that are significantly different from the vehicle control. Where indicated, S9 fraction from the livers of Aroclor 1254-treated rats was included in the incubation at a final concentration of 4.5%. An NADPH-regenerating system is also included to ensure a steady supply of reducing equivalents. The strains used in this study were S. typhimurium TA98 (hisD3052, rfa, uvrB/pKM10 for detection of frame-shift mutations) and S. typhimurium TA100 (hisG45, rfa, uvrB/pKM101 for detection of base-pair substitutions). VCE-003.2 was assessed for its mutagenic potential in the AMES reverse mutation assay. This test was performed in the absence and presence of S9 metabolic activation. VCE-003.2 was found to be negative for genotoxicity in this AMES study. The positive controls all behaved as expected. (PDF 568 kb)

Additional file 5: Effect of oral VCE-003.2 on plasmatic biomarkers. Plasma samples from the indicated groups of animals (*n* = 6) were pooled and subjected to mouse cytokine array (ARY028; R&D Systems) and mouse adipokine array (ARY013; R&D Systems) analysis. The relative expression of the indicated biomarkers is shown. (PDF 338 kb)

Additional file 6: 3-Nitroproprionic acid model of striatal neurodegeneration. 16-week- old C57BL/6 N male mice (Harlan Ibérica, Barcelona, Spain) were subjected to seven intraperitoneal (i.p.) injections of 50 mg/kg 3-NP (one injection each every 12 h prepared in phosphate-buffered saline (PBS)]. 3-NP-treated animals and their respective non-lesioned controls (injected with PBS) were used for pharmacological studies with VCE-003.2. Treatments consisted of oral gavage every 24 h with VCE-003.2 at a dose of 10 mg/kg using sesame oil as vehicle. 12 h after the last injection of 3-NP motor activity, hindlimb clasping and kyphosis were evaluated as previously described [12]. Animals were euthanized at the indicated time after huntingtin-AAV infection or 12 h after the last injection of 3NP and their brains removed. Statistical analysis: One-way ANOVA followed by the Tuckey’s post hoc test was used to determine the statistical significance. All the *in vivo* data were expressed as mean ± SEM. Kruskal-Wallis test was used to determine the statistical in the case of non-parametric analysis. a) Hindlimb Clasping (F = 8.069; *p* = 0.0047) post hoc test: *p* = 0.0036 3NP vs Veh; Locomotor activity (F = 18.62; *p* = 0.0001) post hoc test *p* < 0.0001 3NP vs Veh, *p* = 0.0027 3NP + VCE-003.2 vs 3NP; Kyphosis (F = 28.24 *p* < 0.0001) post hoc test: *p* < 0.0001 3NP vs Veh, *p* < 0.0001 3NP + VCE-003.2 vs 3NP. b) TNF-α (F = 18.17 *p* = 0.0028) post hoc test: *p* = 0.0027 3NP vs Veh, *p* = 0.0138 3NP + VCE-003.2 vs 3NP. IL-6 (Kruskal-Wallis statistic = 6.880 *p* = 0.0071) post hoc test: *p* = 0.0265 3NP vs Veh. c) Average number of neurons per field (F = 15.69 *p* = 0.0012) post hoc test: *p* = 0.0011 3NP vs Veh, *p* = 0.0086 3NP + VCE-003.2 vs 3NP; Number of Iba1^+^ cells (F = 10.82 *p* = 0.0040) post hoc test: *p* = 0.0101 3NP vs Veh, *p* = 0.0059 3NP + VCE-003.2 vs 3NP. (PDF 325 kb)

## Abbreviations

AAV: Adeno associated virus
CBG: Cannabigerol
HD: Huntington’s disease
Htt: Huntingtin
MSN: Medium spiny neuron
PPAR: Peroxisome proliferator-activated receptor
SVZ: Subventricular zone
