# Supplementary material for: Oral administration of the cannabigerol derivative VCE-003.2 promotes subventricular zone neurogenesis and protects against mutant huntingtin-induced neurodegeneration
Source: Transl Neurodegener. 2019 Mar 8;8:9. doi: 10.1186/s40035-019-0148-x (PMC6407204; doi:10.1186/s40035-019-0148-x)
Supplement: Supplementary file 1 — Microsomal Metabolic Stability. Pooled human liver microsomes (final protein concentration 0.5 mg/mL), 0.1 M phosphate buffer pH 7.4 and the test compounds (VCE-003.2, CBG, verapamil and dextromethorphan) were pre-incubated at 37 °C prior to the addition of 1 mM NADPH to initiate the reaction. The final incubation volume was 25 μL. Each compound was incubated for 0, 5, 15, 30 and 45 min. The control (minus NADPH) was incubated for 45 min only. The reactions were stopped by the addition of 50 μL methanol containing internal standard at the appropriate time points. The incubation plates were centrifuged at 2500 rpm for 20 min at 4 °C to precipitate the protein. Following protein precipitation, the sample supernatants were analyzed using LC-MS/MS. From a plot of ln peak area ratio (compound peak area/internal standard peak area) against time, the gradient of the line was determined. Subsequently, half-life and intrinsic clearance was calculated using the equations below: Elimination rate constant (k) = (− gradient). Half-life (t1/2) (min) = $\frac{0.693}{k}$0.693k. Intrinsic Clearance (CLint) (μL/min/mg protein) = $\frac{V\times 0.693}{t_{1/2}}$V×0.693t1/2. where V=Incubation volume mL/mg microsomal protein. (PDF 373 kb) [file 40035_2019_148_MOESM1_ESM.pdf]

Additional File 1. Microsomal Metabolic Stability.

Metabolic Stability (Species = Human)			
**Compound**	**CL_{int} (μL/min/mg protein)**	**SE CL_{int}**	**$T_{1/2}$ (min)**
**Dextrom Etorphan**	23.9	2.21	58.1
**Verapamil**	171	2.13	8.11
**VCE-003.2**	131	8.09	10.6
**CBG**	103	3.19	13.4
