# Supplementary material for: Oral administration of the cannabigerol derivative VCE-003.2 promotes subventricular zone neurogenesis and protects against mutant huntingtin-induced neurodegeneration
Source: Transl Neurodegener. 2019 Mar 8;8:9. doi: 10.1186/s40035-019-0148-x (PMC6407204; doi:10.1186/s40035-019-0148-x)
Supplement: Supplementary file 2 — Cytochrome P450 Inhibition (IC50 Determination). CYP1A Inhibition. VCE-003.2 (0.1, 0.25, 1, 2.5, 10, 25 μM in DMSO; final DMSO concentration = 0.3%) was incubated with human liver microsomes (0.25 mg/mL) and NADPH (1 mM) in the presence of the probe substrate ethoxyresorufin (0.5 μM) for 5 min at 37 °C. The selective CYP1A inhibitor, alpha-naphthoflavone, was screened alongside the test compounds as a positive control. CYP2B6 Inhibition. VCE-003.2 (0.1, 0.25, 1, 2.5, 10, 25 μM in DMSO; final DMSO concentration = 0.3%) was incubated with human liver microsomes (0.1 mg/mL) and NADPH (1 mM) in the presence of the probe substrate bupropion (110 μM) for 5 min at 37 °C. The selective CYP2B6 inhibitor, ticlopidine, was screened alongside the test compounds as a positive control. CYP2C8 Inhibition. VCE-003.2 (0.1, 0.25, 1, 2.5, 10, 25 μM in DMSO; final DMSO concentration = 0.3%) was incubated with human liver microsomes (0.25 mg/mL) and NADPH (1 mM) in the presence of the probe substrate paclitaxel (7.5 μM) for 30 min at 37 °C. The selective CYP2C8 inhibitor, montelukast, was screened alongside the test compounds as a positive control. CYP2C9 Inhibition. VCE-003.2 (0.1, 0.25, 1, 2.5, 10, 25 μM in DMSO; final DMSO concentration = 0.3%) was incubated with human liver microsomes (1 mg/mL) and NADPH (1 mM) in the presence of the probe substrate tolbutamide (120 μM) for 60 min at 37 °C. The selective CYP2C9 inhibitor, sulphaphenazole, was screened alongside the test compounds as a positive control. CYP2C19 Inhibition. VCE-003.2 (0.1, 0.25, 1, 2.5, 10, 25 μM in DMSO; final DMSO concentration = 0.3%) was incubated with human liver microsomes (0.5 mg/mL) and NADPH (1 mM) in the presence of the probe substrate mephenytoin (25 μM) for 60 min at 37 °C. The selective CYP2C19 inhibitor, tranylcypromine, was screened alongside the test compounds as a positive control. CYP2D6 Inhibition. VCE-003.2 (0.1, 0.25, 1, 2.5, 10, 25 μM in DMSO; final DMSO concentration = 0.3%) was incubated with h [file 40035_2019_148_MOESM2_ESM.pdf]

Additional File 2. Cytochrome P450 Inhibition (IC50 Determination).

Cytochrome P450 Inhibition (Isoform)	IC50 (µM)
**CYP1A, Substrate = Etoxyresorufin**	>25
**CYP2B6, Substrate = Bupropion**	>25
**CYP2C8, Substrate = Paclitaxel**	13.0 ± 2.4
**CYP3A4, Substrate = Testosterone**	>25
**CYP2C19, Substrate = Mephenytoin**	>25
**CYP2D6, Substrate = Dextromethorphan**	>25
**CYP3A4, Substrate = Midazolam**	>25
**CYP2C9, Substrate = Tolbutamide**	15.7 ± 1.96
