# Supplementary material for: Oral administration of the cannabigerol derivative VCE-003.2 promotes subventricular zone neurogenesis and protects against mutant huntingtin-induced neurodegeneration
Source: Transl Neurodegener. 2019 Mar 8;8:9. doi: 10.1186/s40035-019-0148-x (PMC6407204; doi:10.1186/s40035-019-0148-x)
Supplement: Supplementary file 3 — hERG Channel Inhibition (IC50 Determination). The experiments were performed on an IonWorks™ HT instrument (Molecular Devices Corporation), which automatically performs electrophysiology measurements in 48 single cells simultaneously in a specialized 384-well plate (PatchPlate™). The cells used were Chinese hamster ovary (CHO) cells stably transfected with hERG (cell-line obtained from Cytomyx, UK). A single-cell suspension was prepared in extracellular solution (Dulbecco’s phosphate buffered saline with calcium and magnesium pH 7–7.2) and aliquots added to each well of a PatchPlate™. Cells were positioned over a small hole at the bottom of each well by applying a vacuum beneath the plate to form an electrical seal. The resistance of each seal was measured via a common ground-electrode in the intracellular compartment and individual electrodes placed into each of the upper wells. Electrical access to the cell was achieved by circulating a perforating agent, amphotericin, underneath the PatchPlate™ and then measuring the pre-compound hERG current. An electrode is positioned in the extracellular compartment and a holding potential of − 80 mV applied for 15 s. The hERG channels were then activated by applying a depolarizing step to + 40 mV for 5 s and then clamped at − 50 mV for 4 s to elicit the hERG tail current, before returning to − 80 mV for 0.3 s. VCE-003.2 were added to the upper wells of the PatchPlate™. Solutions were prepared by diluting 10 mM DMSO solutions of the test compound into extracellular buffer such that the final concentrations tested are 0.008, 0.04, 0.2, 1, 5 and 25 μM (final DMSO concentration 0.25%). Quinidine, an established hERG inhibitor, was included as a positive control and buffer containing 0.25% DMSO was included as a negative control. Post-compound currents were then expressed as a percentage of pre-compound currents and plotted against concentration for each compound. Where concentration-dependent inhibition is observed, the data are [file 40035_2019_148_MOESM3_ESM.pdf]

Additional File 3. hERG Channel Inhibition (IC_{50} Determination).

hERG Channel Inhibition	
**Compound**	**IC50 (µM)**
**VCE-003.2**	>25
**Quinidine**	1,76 ± 0.382
