# Supplementary material for: Oral administration of the cannabigerol derivative VCE-003.2 promotes subventricular zone neurogenesis and protects against mutant huntingtin-induced neurodegeneration
Source: Transl Neurodegener. 2019 Mar 8;8:9. doi: 10.1186/s40035-019-0148-x (PMC6407204; doi:10.1186/s40035-019-0148-x)
Supplement: Supplementary file 4 — AMES Data Summary. AMES Experimental Procedure. Approximately ten million bacteria are exposed in triplicate to VCE-003-.2 (7.8, 15.6, 31.3, 62.5, 125 and 250 μg/ml), a negative control (vehicle) and a positive control for 90 min in medium containing a low concentration of histidine (sufficient for about 2 doublings). The cultures were then diluted into indicator medium lacking histidine and dispensed into 48 wells of a 384 well plate (micro-plate format, MPF). The plate was incubated for 48 h at 37 °C, and the number of wells showing growth were counted and compared to the vehicle control. An increase in the number of colonies of at least two-fold over baseline (mean + SD of the vehicle control) and a dose response indicates a positive response. An unpaired, one-sided Student’s T-test was used to identify conditions that are significantly different from the vehicle control. Where indicated, S9 fraction from the livers of Aroclor 1254-treated rats was included in the incubation at a final concentration of 4.5%. An NADPH-regenerating system is also included to ensure a steady supply of reducing equivalents. The strains used in this study were S. typhimurium TA98 (hisD3052, rfa, uvrB/pKM10 for detection of frame-shift mutations) and S. typhimurium TA100 (hisG45, rfa, uvrB/pKM101 for detection of base-pair substitutions). VCE-003.2 was assessed for its mutagenic potential in the AMES reverse mutation assay. This test was performed in the absence and presence of S9 metabolic activation. VCE-003.2 was found to be negative for genotoxicity in this AMES study. The positive controls all behaved as expected. (PDF 568 kb) [file 40035_2019_148_MOESM4_ESM.pdf]

Additional File 4. AMES Data Summary.

Compound	Test Strain	S9	AMES Result	Highest Conc. Tested*	Comment
**2-nitrofluorine + 4-nitroquinoline N-oxide**	TA98	no	Positive	4 µg/ml + 2 µg/ml	positive control
**2-nitrofluorine + 4-nitroquinoline N-oxide**	TA100	no	Positive	4 µg/ml + 2 µg/ml	positive control
**aminoanthracene**	TA98	yes	Positive	5 µg/ml	positive control
**aminoanthracene**	TA100	yes	Positive	5 µg/ml	positive control
**VCE-003.2**	TA98	no	Negative	250µg/mL	
	TA100	no	Negative	250µg/mL	
	TA98	yes	Negative	250µg/mL	
	TA100	yes	Negative	250µg/mL	

*Highest soluble concentration of test article in Ames assay conditions.
