# Supplementary figures and images for: Oral administration of the cannabigerol derivative VCE-003.2 promotes subventricular zone neurogenesis and protects against mutant huntingtin-induced neurodegeneration
Source: Transl Neurodegener. 2019 Mar 8;8:9. doi: 10.1186/s40035-019-0148-x (PMC6407204; doi:10.1186/s40035-019-0148-x)

Additional file 5. Effect of oral VCE-003.2 on plasmatic biomarkers.

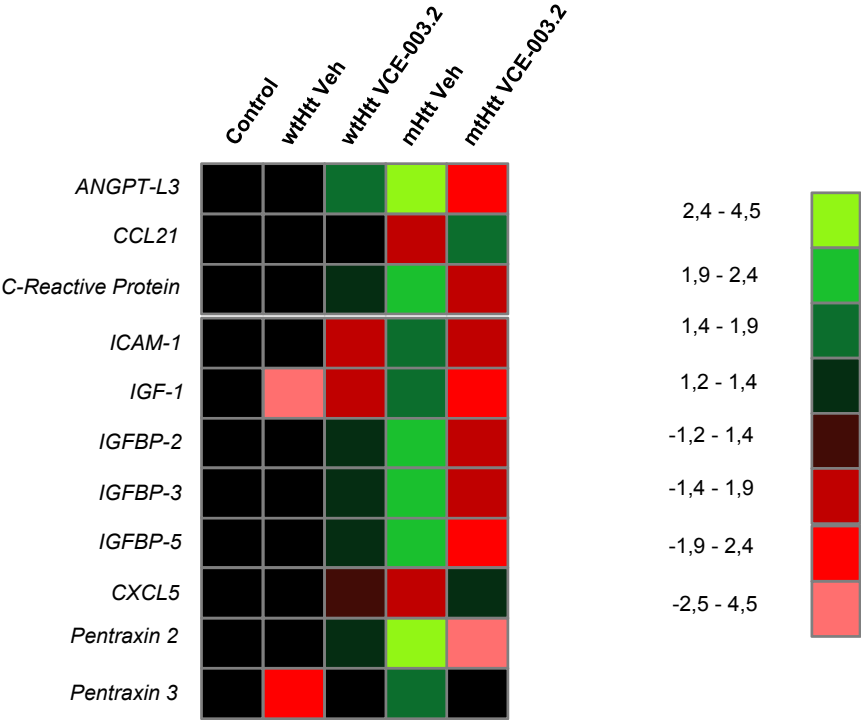

Supplement: Supplementary file 5 — Effect of oral VCE-003.2 on plasmatic biomarkers. Plasma samples from the indicated groups of animals (n = 6) were pooled and subjected to mouse cytokine array (ARY028; R&D Systems) and mouse adipokine array (ARY013; R&D Systems) analysis. The relative expression of the indicated biomarkers is shown. (PDF 338 kb) [file 40035_2019_148_MOESM5_ESM.pdf]

**Additional File 6. 3-Nitropropionic acid model of striatal neurodegeneration.**

**a**

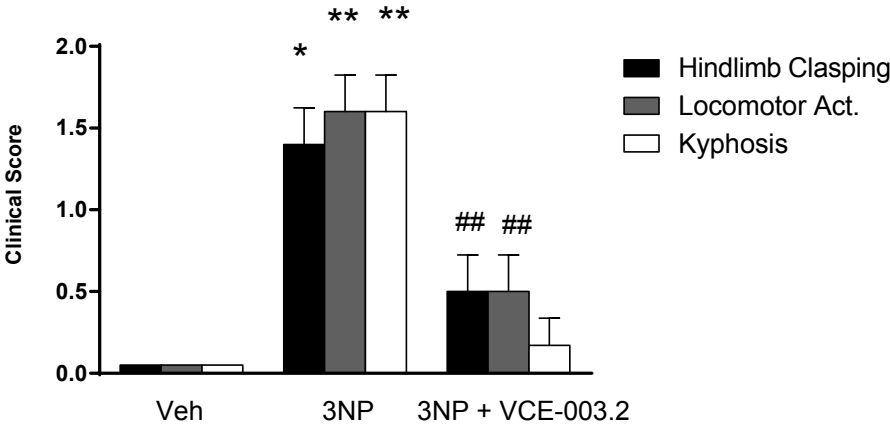

**b**

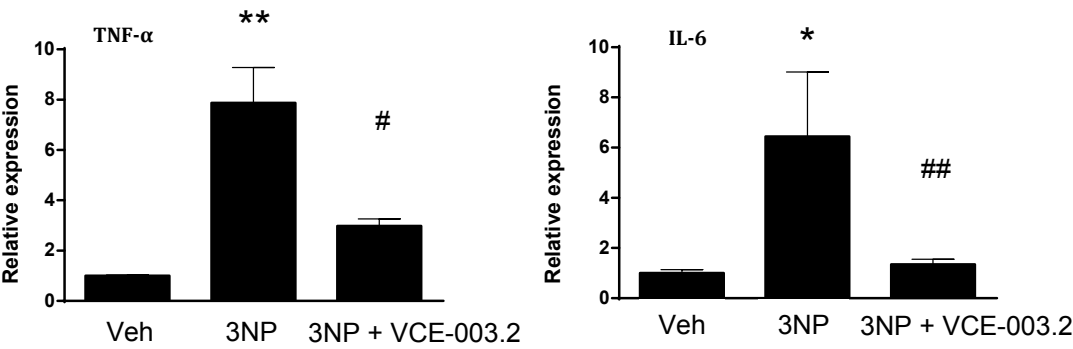

**c**

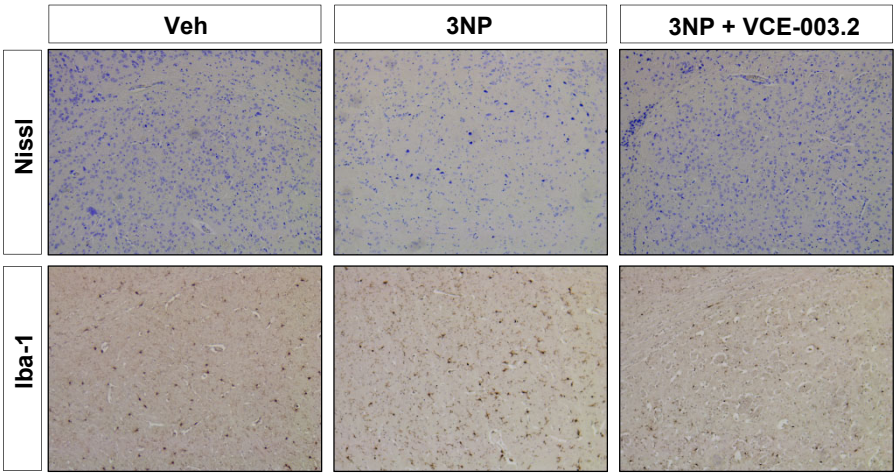

Supplement: Supplementary file 6 — 3-Nitroproprionic acid model of striatal neurodegeneration. 16-week- old C57BL/6 N male mice (Harlan Ibérica, Barcelona, Spain) were subjected to seven intraperitoneal (i.p.) injections of 50 mg/kg 3-NP (one injection each every 12 h prepared in phosphate-buffered saline (PBS)]. 3-NP-treated animals and their respective non-lesioned controls (injected with PBS) were used for pharmacological studies with VCE-003.2. Treatments consisted of oral gavage every 24 h with VCE-003.2 at a dose of 10 mg/kg using sesame oil as vehicle. 12 h after the last injection of 3-NP motor activity, hindlimb clasping and kyphosis were evaluated as previously described [12]. Animals were euthanized at the indicated time after huntingtin-AAV infection or 12 h after the last injection of 3NP and their brains removed. Statistical analysis: One-way ANOVA followed by the Tuckey’s post hoc test was used to determine the statistical significance. All the in vivo data were expressed as mean ± SEM. Kruskal-Wallis test was used to determine the statistical in the case of non-parametric analysis. a) Hindlimb Clasping (F = 8.069; p = 0.0047) post hoc test: p = 0.0036 3NP vs Veh; Locomotor activity (F = 18.62; p = 0.0001) post hoc test p < 0.0001 3NP vs Veh, p = 0.0027 3NP + VCE-003.2 vs 3NP; Kyphosis (F = 28.24 p < 0.0001) post hoc test: p < 0.0001 3NP vs Veh, p < 0.0001 3NP + VCE-003.2 vs 3NP. b) TNF-α (F = 18.17 p = 0.0028) post hoc test: p = 0.0027 3NP vs Veh, p = 0.0138 3NP + VCE-003.2 vs 3NP. IL-6 (Kruskal-Wallis statistic = 6.880 p = 0.0071) post hoc test: p = 0.0265 3NP vs Veh. c) Average number of neurons per field (F = 15.69 p = 0.0012) post hoc test: p = 0.0011 3NP vs Veh, p = 0.0086 3NP + VCE-003.2 vs 3NP; Number of Iba1+ cells (F = 10.82 p = 0.0040) post hoc test: p = 0.0101 3NP vs Veh, p = 0.0059 3NP + VCE-003.2 vs 3NP. (PDF 325 kb) [file 40035_2019_148_MOESM6_ESM.pdf]
